# TAR DNA Binding Protein-43 Loss of Function Induced by Phosphorylation at S409/410 Blocks Autophagic Flux and Participates in Secondary Brain Injury After Intracerebral Hemorrhage

**DOI:** 10.3389/fncel.2018.00079

**Published:** 2018-03-22

**Authors:** Liang Sun, Kai Zhang, Weiwei Zhai, Haiying Li, Haitao Shen, Zhengquan Yu, Gang Chen

**Affiliations:** ^1^Department of Neurosurgery, Huai’an First People’s Hospital, Nanjing Medical University, Huai’an, China; ^2^Department of Neurosurgery & Brain and Nerve Research Laboratory, The First Affiliated Hospital of Soochow University, Suzhou, China

**Keywords:** TDP-43, nucleus loss, mTOR, dynactin1, autophagic flux, intracerebral hemorrhage, calcineurin

## Abstract

This study aimed to determine the role of TAR DNA binding protein-43 (TDP-43) in intracerebral hemorrhage (ICH)-induced secondary brain injury (SBI) and its underlying mechanisms. After ICH, expression of TDP-43 in the nucleus was significantly decreased, and its expression in the cytoplasm increased both *in vivo* and *in vitro*, which indicates that TDP-43 translocates from the nucleus to the cytoplasm during SBI after ICH. In addition, mutations at S409/410 of TDP-43 could inhibit its phosphorylation, attenuate nuclear loss, and abolish the increase in neuronal apoptosis in the subcortex. Inhibition of TDP-43 phosphorylation attenuated ICH-induced downregulation of mTOR activity and dynactin1 expression, which may relieve blocking of autophagosome-lysosome fusion and the increase of autophagosomal and lysosomal biogenesis induced by ICH. However, knockdown of TDP-43 could worsen ICH-induced SBI. Furthermore, TDP-43 could be dephosphorylated by calcineurin (CN), and CN activity was increased by OxyHb treatment. In conclusion, this study demonstrated that TDP-43 loss-of-function by phosphorylation at S409/410 may block autophagosome-lysosome fusion and induce elevation of LC3II and p62 levels by inhibiting the activity of mTOR and expression of dynactin1. This mechanism may play an important role in ICH-induced SBI, and TDP-43 may be a potential therapeutic target.

## Introduction

Intracranial hemorrhage (ICH) is considered the most fatal subtype of stroke. It has a high mortality and accounts for 10%–15% of stroke cases (Chen et al., 2014; Leclerc et al., 2016; Zhai et al., 2016; Mao et al., 2017). There are many causes of ICH, and hypertension is a major factor for Asians. A hematoma can directly destroy tracts, which results in dysfunctions of language and physical activity and can be life threatening. A series of pathophysiological processes after ICH, which includes development of brain edema, activation of apoptotic programs, ischemia of brain tissue surrounding the hematoma, and toxic effects of extracellular heme, are referred to as secondary brain injury (SBI). These processes may be subjected to intervention (Xi et al., 2006; Aronowski and Zhao, 2011; Wu et al., 2016; Jiang et al., 2017).

TAR DNA binding protein-43 (TDP-43) is a multifunctional DNA and RNA binding protein that participates in RNA transcription, alternative splicing, and regulation of mRNA stability in cells (Chang et al., 2016; Le et al., 2016; Schwenk et al., 2016). TDP-43 has been identified as a key component of the pathological substrate of neuronal and glial inclusions in patients with amyotrophic lateral sclerosis (ALS) and frontotemporal lobar degeneration (FTLD). Recent reports have shown that TDP-43 is a nuclear protein that can translocate from the nucleus to the cytoplasm to form ubiquitin-positive inclusions (UPIs) that are neurotoxic in ALS and FTLD (Porta et al., 2015; Halliday et al., 2016; Mashiko et al., 2016). Moreover, extracellular TDP-43 aggregates can target the MAPK/MAK/MRK overlapping kinase (MOK) pathway and trigger caspase-3/IL-18 signaling in microglia, which results in ALS and FTLD (Leal-Lasarte et al., 2017). In addition, redistribution of TDP-43 can induce neural apoptosis (Suzuki et al., 2011), which may play a key role during SBI after ICH.

Numerous recent studies have shown that TDP-43 can undergo a number of posttranslational modifications including proteolytic cleavage (generating low-molecular-weight 25 and 35 kDa species), phosphorylation, and ubiquitination. Phosphorylated TDP-43 plays important roles in formation of UPIs in FTLD and ALS (Zhang et al., 2007; Li et al., 2015; Xia et al., 2016; Yamashita et al., 2016). Phosphorylation of TDP-43 by CK1 may be involved in accumulation of the protein. It has been reported that there are at least five phosphorylation sites in the entire TDP-43 fragment, and S409/410 is thought to be the major one (Hasegawa et al., 2008; Neumann et al., 2009; Liachko et al., 2013).

In recent years, evidence has indicated that loss of TDP-43 in the nucleus enhances global gene expression in the autophagy-lysosome pathway (ALP) and increases autophagosomal and lysosomal biogenesis. In addition, loss of TDP-43 downregulates dynactin1 to impair fusion of autophagosomes with lysosomes (Xia et al., 2016). It is now well established that TDP-43 plays an important role in ALP (Sullivan et al., 2016) and neuronal apoptosis (Estes et al., 2011). However, the effects of TDP-43 phosphorylation on ALP are unclear, and the corresponding roles of TDP-43 in ICH are unknown.

To elucidate mechanisms underlying effects of TDP-43 on neuronal apoptosis after ICH, Suzuki et al. (2011) have suggested that preventing redistribution of TDP-43 may reduce neural apoptosis. Thus, we hypothesized that TDP-43 phosphorylation would induce its nuclear mislocalization and loss of function, resulting in ALP block and SBI induction.

## Materials and Methods

### Antibodies

Anti-TDP-43 (12892-1-AP) was provided by Novus Biologicals (Littleton, CO, USA). Anti-mTOR (ab32028), anti-dynactin1 (DCTNI; ab11806), anti-p62 (ab56416), anti-P-TDP43 (phospho S409 + S410), anti-NeuN (ab104224), and anti-histone H3 (ab1791) were purchased from Abcam (Cambridge, MA, USA). Anti-β-tubulin (sc-5274) was purchased from Santa Cruz (Santa Cruz, CA, USA).

### Experimental Animals

Adult male Sprague-Dawley rats weighing 280–300 g and pregnant rats were purchased from the Laboratory Animal Center, Medical College of Soochow University, Suzhou, Jiangsu, China. The animal experimental agreements were approved by the Experimental Animals Committee of the First Affiliated Hospital of Soochow University and complied with the ARRIVE guidelines. All rats were housed in a quiet environment maintained at 18–22°C with stable humidity and animals had free access to food and water. We strived to minimize the number of rats used and their pain.

### ICH Modeling in Rats

**Figure 1:**
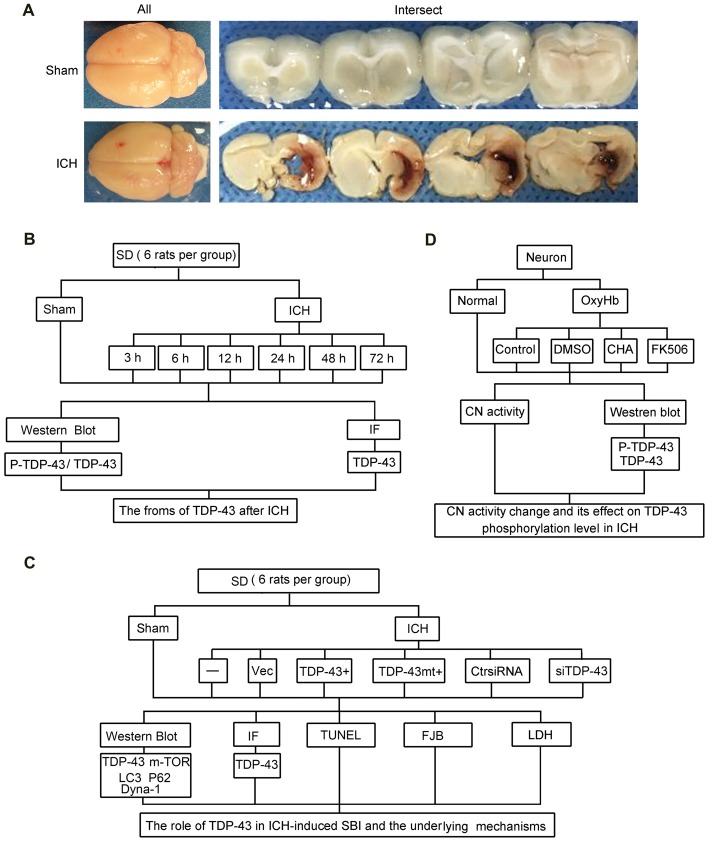
Intracerebral hemorrhage (ICH) model and experimental design. **(A)** Gross morphology of the specimens from the ICH model. **(B)** Time course of protein levels of TAR DNA binding protein-43 (TDP-43) and phosphorylation of TDP-43 after ICH. A total of 42 rats survived the surgery (50 rats were used) and were randomly assigned to seven groups (six rats per group): sham group and six experimental groups arranged by time points of 3 h, 6 h, 12 h, 24 h, 48 h and 72 h after ICH. At the appropriate time point, rats were killed and the right hemisphere basal ganglia tissues (cerebral hemorrhage and surrounding areas) were dissected and collected for analysis. **(C)** Effects of TDP-43 on ICH-induced secondary brain injury (SBI) and its potential mechanisms. A total of 63 rats (75 rats were used, 63 rats survived) were randomly divided into seven groups: sham, ICH, ICH + empty vector, ICH + TDP-43 plasmid, ICH + TDP-43 plasmid mutation and ICH + TDP-43 siRNA (nine rats per group). After the indicated treatments, rats were killed, and the right hemisphere basal ganglia tissues (cerebral hemorrhage and surrounding areas) were dissected and collected for analysis. **(D)** Mechanisms underlying dephosphorylation of TDP-43 after ICH. Extracted neurons were transfected with negative control siRNA, followed by DMSO, chlorogenic acid (CHA), and FK506 treatment. Next, 48 h after transfection, cells were exposed to 10 μM OxyHb for an additional 48 h to mimic ICH conditions. Then, cells were harvested for western blots and determination of calcineurin (CN) activity.

Rat (280–300 g) were fully anesthetized and then fixed in a stereotaxic apparatus frame (Shanghai Ruanlong Science and Technology Development Co., Ltd., Shanghai, China). The rats were then placed supine on a heating pad that was maintained at approximately 27–35°C. The experimental ICH model was produced using stereotaxic insertion of a needle with a rounded tip and a side hole into the basal ganglia. The position of the basal ganglia was 3.5 mm lateral to the midline, 0.2 mm posterior to Bregma, and 5.5 mm ventral to the cortical surface. After the microinjector was in position, autologous blood was injected over 5 min, and the needle was left in place for an additional 5 min (Hu et al., 2011). Bone wax was used to block the burr hole to prevent loss of cerebrospinal fluid and blood from the midline vessels. Next, we sutured the scalp and returned the rat to its cage where it had free access to food and water. Representative brain slices from the ICH model are shown in Figure 1A.

### Experimental Design

The experiments were divided into three parts including *in vivo* and *in vitro* experiments. In part 1, 42 rats survived surgery (50 rats were used) and were randomly assigned to seven groups (six rats per group): sham group and six experimental groups arranged by time points of 3 h, 6 h, 12 h, 24 h, 48 h and 72 h after ICH. At the appropriate time point, rats were killed, and the right hemisphere basal ganglia tissues (cerebral hemorrhage and surrounding areas) were separated and collected for analysis (Figure 1B). In part 2, 63 rats (75 rats were used, 63 rats survived) were randomly divided into seven groups: sham group, ICH group, ICH + empty vector group, ICH + TDP-43 plasmid group, ICH + TDP-43 plasmid mutation group, and ICH + TDP-43 siRNA group (nine rats per group). After the indicated treatments, rats were killed, and the right hemisphere basal ganglia tissues (cerebral hemorrhage and surrounding areas) were separated and collected for analysis. Transfection of siRNA and plasmid in the rat brain was performed 48 h before onset of ICH. At 48 h after ICH, which was chosen base on results of the first experiment, the brain cortices of nine rats were dissected for terminal deoxynucleotidyltransferase-mediated dUTP nick end labeling (TUNEL) staining, Fluoro-Jade B (FJB) staining, immunofluorescence staining, and western blot assay (Figure 1C). In part 3, cultured neurons were transfected with negative control siRNA, followed by DMSO, chlorogenic acid (CHA), and FK506 treatment. Next, 48 h after transfection, cells were stimulated with 10 μM OxyHb for an additional 48 h to mimic ICH conditions. Then cells were harvested for western blots and determination of calcineurin (CN) activity (Figure 1D).

### Cell Cultures and Treatments

Whole brains of 17-day-old rat embryos were used to prepare primary neuron-enriched cultures. We tried to minimize the number of embryos used and their suffering. In brief, we removed blood vessels and the meninges, and then the brains were digested with 0.25% trypsin for 5 min. Next, we centrifuged the brain suspension at 500× *g* for 5 min and inoculated neuronal cells into 6-well and 12-well plates in Neurobasal Medium (GIBCO, Carlsbad, CA, USA). Neurons were maintained in a 5% CO_2_ incubator at 37°C. Half of the culture medium was replaced every 2 days for 1 week. Cells were then transfected with siRNA. To mimic ICH and evaluate effects of TDP-43 *in vitro*, cells were exposed to OxyHb at a concentration of 10 μM. After these treatments, cellular morphology was observed using an inverted phase contrast microscope, and total cell protein was collected and stored at −80°C until tested.

### Nuclear Protein Extraction

Nuclear protein extraction was performed using a Nuclear and Cytoplasmic Protein Extraction Kit (P0027, Beyotime) according to the manufacturer’s instructions.

### Western Blot Analysis

Brain tissues around the hematoma, which had been stored in liquid nitrogen, were thoroughly ground on ice. RIPA lysis buffer (Beyotime) was used to lyse the brain tissue for 30 min. The lysates were then transferred to a centrifuge tube and centrifuged at 12,000 *g* for 10 min at 4°C. The supernatant was collected, and a standard BCA method (P0012, Beyotime) was used to determine protein concentration. Then, protein samples (100 mg/lane) were loaded onto a 10% SDS polyacrylamide gel, separated and electrophoretically transferred to a PVDF membrane (IPVH00010, Millipore, Billerica, MA, USA). The membrane was then blocked with 5% nonfat milk for 2 h at 37°C. Next, the membrane was incubated with the primary antibody overnight at 4°C, followed by incubation with the horseradish peroxidase-linked secondary antibody for 1.5 h at 37°C. The membrane was washed with PBST and visualized using enhanced chemiluminescence detection (3100 Mini, Clinx Science Instruments Co.). Relative quantities of protein levels were analyzed using ImageJ software.

### Immunofluorescence Microscopy

We performed double labeling for TDP-43 and NeuN to assess expression of TDP-43 in neurons. The rat brain samples were fixed in 4% paraformaldehyde and embedded in paraffin. Next, sections were incubated with the primary antibody (TDP-43, 1:100) overnight at 4°C followed by incubation with the NeuN antibody (neuronal cell marker, 1:100) overnight at 4°C. Then, sections were incubated with the secondary antibodies, which included Alexa Fluor 488 donkey anti-rabbit IgG antibody, Alexa Fluor 555 donkey anti-mouse IgG antibody, Alexa Fluor 488 donkey anti-mouse IgG antibody, and Alexa Fluor 555 donkey anti-rabbit IgG antibody (Life Technologies, Carlsbad, CA, USA, 1:300). Normal rabbit IgG and normal mouse IgG were used as negative controls (data not shown). Finally, sections were observed using a fluorescence microscope (Olympus BX50/BX-FLA/DP70, Olympus Co., Japan), and relative fluorescence intensity was analyzed using ImageJ software.

### siRNAs and Plasmid Construction

Specific siRNAs against TDP-43 were provided by Ribobio. Knockdown efficiency of siRNAs was determined by transfection *in vitro* and detection by western blots. The most efficient siRNAs were used in this study, and the TDP-43 target sequences were as follows:

1. GAGAGGACTTGATCATTAA
2. CAGCGTGCATATATCCAAT
3. TGCTGAACCTAAGCATAAT

The coding region of rat TDP-43 cDNA was subcloned into a pEGFP-N2 expression vector to produce the pEGFPN2-TDP-43 construct (without an EGFP tag). In addition, a rat TDP-43 cDNA construct with mutations at a possible key phosphorylation site (S409/410A mutant: Ser409/410 were changed to alanine) was also subcloned into a pEGFP-N2 expression vector (without an EGFP tag). All constructs were confirmed by DNA sequencing.

### Transfection of siRNA in the Rat Brain

Transfection of siRNA in the rat brain was done using Entranster-*in vivo* RNA transfection reagent (18668-11-1 Engreen) according to the manufacturer’s instructions. Briefly, 5 nmol TDP-43 siRNA and 5 nmol scramble siRNA were dissolved in 66.5 μL DEPC RNase-free water. Then, 5 μL Entranster-*in vivo* RNA transfection reagents and 5 μL normal saline were added immediately to 10 μL siRNA or 10 μL scramble siRNA. The solution was mixed for 15 min at room temperature. Finally, 20 μL Entranster-siRNA mixture was injected intracerebroventricularly 48 h before ICH.

### Transfection of Plasmid in the Rat Brain

Transfection of plasmid in the rat brain was performed using Entranster-*in vivo* DNA transfection reagent (18668-11-2 Engreen) according to the manufacturer’s instructions. Briefly, 10 μL Entranster-*in vivo* DNA transfection reagent was added to 5 μg plasmid or 5 μg empty vector. The solution was mixed for 15 min at room temperature. Finally, 15 μL Entranster-*in vivo*-plasmid mixture was injected intracerebroventricularly 48 h before ICH.

### Transfection of siRNA and Plasmid in Cultured Neurons

Transfection of siRNA and plasmid in cultured neurons was performed using Lipofectamine^®^ 3000 Transfection Reagent (Invitrogen, Grand Island, NY, USA) according to the manufacturer’s instructions. Neurons were transfected with TDP-43 siRNA, empty vector, TDP-43 plasmid, or scramble siRNA. Next, 48 h after transfection, neurons were exposed to 10 μM OxyHb for an additional 48 h. After the indicated treatments, neurons were harvested for further analysis.

### TUNEL Staining

Brain tissues embedded in paraffin were used for TUNEL staining. The sections were deparaffinized, dehydrated by heating at 75°C in an oven for 60 min, and then rehydrated through xylenes and graded ethanol solutions to water. The sections were then incubated in Triton X-100 for 10 min. After washing three times in PBS (5 min per wash), sections were incubated with the TUNEL reaction mixture for 1 h at 37°C. The sections were washed again three times in PBS (5 min per wash). After the final wash, sections were coverslipped with an anti-fading mounting medium containing DAPI. The number of TUNEL-positive neurons per millimeter was determined for each sample. Cell counts from each brain sample were averaged to provide a mean value.

### FJB Staining

Cell necrosis in brain tissue was assessed using FJB. FJB procedures were identical to those for TUNEL. The sections were deparaffinized, dehydrated in an oven, rehydrated through xylenes and graded ethanol solutions to water, and permeabilized in 0.04% Triton X-100. The sections were then incubated in FJB dye solution and visualized using a fluorescence microscope (Olympus BX50/BXFLA/DP70, Olympus). FJB-positive cells were counted by an observer who was blind to the experimental groups. To evaluate the extent of cell necrosis, the necrotic index was defined as the average number of FJB-positive cells in each section counted in six microscopic fields (400× magnification).

### Assay of Lactate Dehydrogenase (LDH) Activity

Lactate dehydrogenase (LDH) activity in cell culture supernatants was quantified using a specific LDH assay according to the manufacturer’s instructions (Nanjing Jiancheng Bioengineering Institute, Nanjing, China). First, reaction wells, which included standard wells, sample wells, control wells, and empty wells, were set up, and reagents sufficient for the number of assays to be performed were prepared. Then, a master mix of the reaction mix was prepared to ensure consistency of concentration and added to the relative groups. Finally, activity of LDH was measured immediately at OD 450 nm using a microplate reader.

### Analysis of CN Activity

Analysis of CN activity was performed using a CN activity quantification kit (GMS50042.1, Genmed Scientifics, Inc., Wilmington, DE, USA) according to the manufacturer’s instructions.

### Statistical Analyses

GraphPad Prism 6 was used for all statistical analysis. Data were analyzed by one-way ANOVA followed by either a Dunnett’s or a Sidak’s *post hoc* test. The former was used for comparisons to a single control group, and the latter was used to compare with the preselected groups. All data are expressed as mean ± SEM. A value of *p* < 0.05 was considered statistically significant.

## Results

### TDP-43 Translocated From the Nucleus to the Cytoplasm After ICH

**Figure 2:**
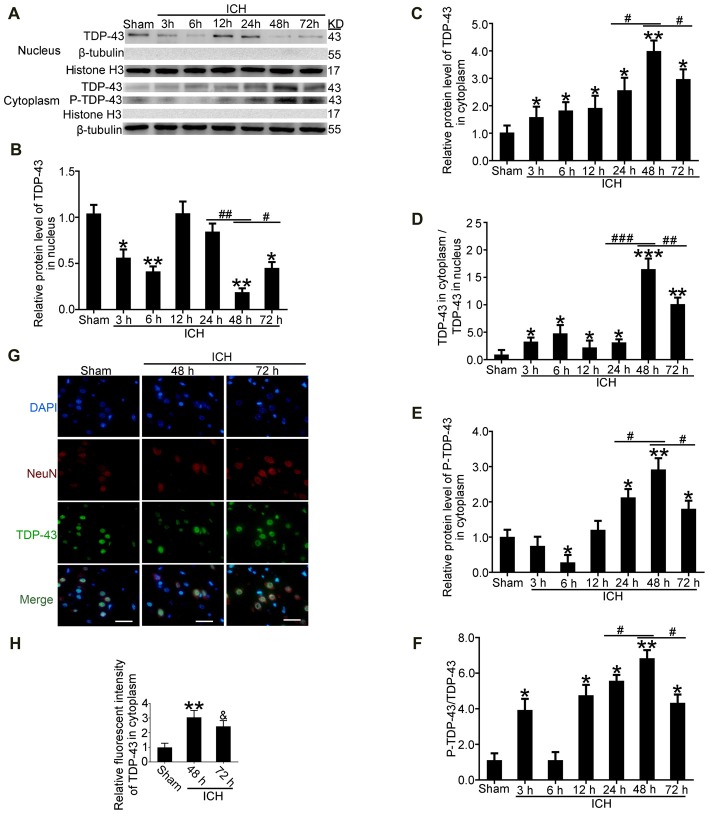
TDP-43 translocated from the nucleus to the cytoplasm after ICH. **(A)**TDP-43 and P-TDP-43 expression levels in the nucleus andcytoplasm were measured by western blot at different times after ICH. **(B,C)** TDP-43 protein levels in the nucleus were quantified, and the mean values of TDP-43 protein levels in the sham group were normalized to 1.0. β-tubulin and histone H3 served as loading controls (all values are mean ± SEM, *p < 0.05, **p < 0.01 vs. sham, ^#^*p* < 0.05 ^##^*p* < 0.01 24 h vs. 48 h, ^#^*p* < 0.05 48 h vs. 72 h), *n* = 6. **(D)** The ratio of TDP-43 in the cytoplasm to that in the nucleus was calculated (all values are mean ± SEM, *p < 0.05, **p < 0.01 vs. sham, ***p < 0.001 vs. sham; ^#^*p* < 0.05, ^##^*p* < 0.01 24 h vs. 48 h, ^###^*p* < 0.001 24 h vs. 48 h, ^#^*p* < 0.05 48 h vs. 72 h), *n* = 6. **(E,F)** P-TDP-43 protein levels in the cytoplasm were quantified, and the mean values of p-TDP-43 protein levels in the sham group were normalized to 1.0. β-tubulin and TDP-43 served as loading controls (all values are mean ± SEM, *p < 0.05, **p < 0.01 vs. sham, ^#^*p* < 0.05 24 h vs. 48 h, ^#^*p* < 0.05 48 h vs. 72 h), *n* = 6. **(G,H)** Immunofluorescence staining to assess changes in TDP-43 at 48 h after ICH *in vivo*. The brain region used for the slides was between the cerebral cortex and the perihematomal. All values are mean ± SEM, **p < 0.01 48 h vs. sham, ^&^*p* < 0.05 48 h vs. 72 h, scale bar: 50 μm.

After induction of ICH, compared with the sham group, western blot analysis showed that protein levels of TDP-43 in the nucleus were significantly decreased starting at 24 h and reached the lowest levels at 48 h. In contrast, protein levels of TDP-43 in the cytoplasm showed an opposite trend and peaked at 48 h (Figures 2A–C). Similarly, the ratio of TDP-43 levels in the cytoplasm to that in the nucleus was significantly increased after ICH and peaked at 48 h (Figure 2D). In addition, phosphorylation of TDP-43 increased gradually with a time course similar to the increase in cytoplasmic TDP-43 after ICH (Figures 2A,E,F). Immunofluorescence staining analysis *in vivo* confirmed that TDP-43 translocated from the nucleus to the cytoplasm, and this shift peaked at 48 h after induction of ICH. The relative fluorescence intensity of TDP-43 was significantly increased at 48 h compared with the sham group, and then it rebounded at 72 h after ICH, which suggests that TDP-43 returns to the nucleus after 48 h (Figures 2G,H).

### TDP-43 Phosphorylation Was Required for Nuclear Loss After ICH

**Figure 3:**
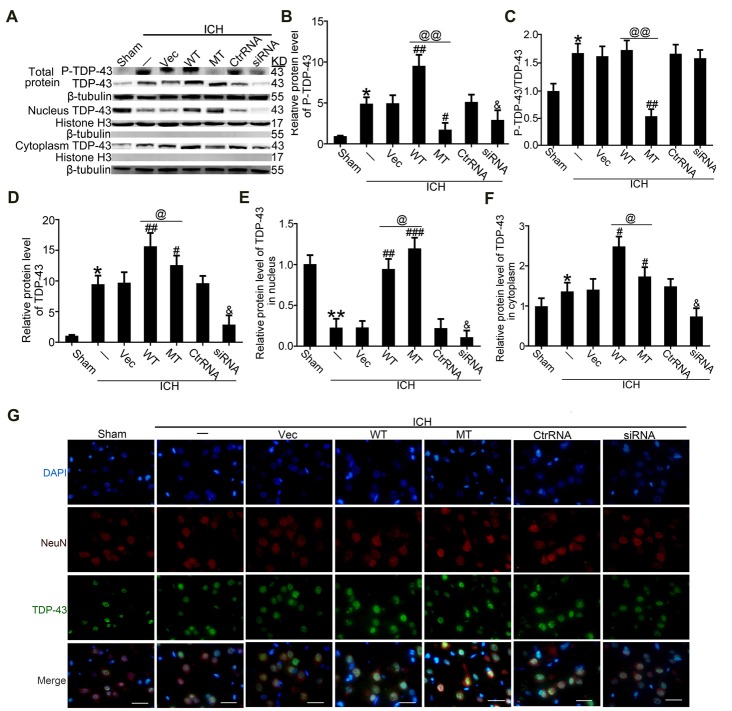
TDP-43 mutationabolished its nuclear loss induced by ICH. **(A)** Western blotanalysis of TDP-43 and P-TDP-43 protein levels after exposureto the TDP-43 plasmid mutation in the whole cell lysate, nucleus and cytoplasm. **(B)** P-TDP-43 protein levels were normalized to that of β-tubulin. **(C)** P-TDP-43 protein levels were normalized to that of TDP-43. **(D)** TDP-43 protein levels were normalized to that of β-tubulin. **(E)** TDP-43 protein levels in the nucleus were quantified, and the mean values of TDP-43 protein levels in the sham group were normalized to 1.0. Histone H3 served as the loading control. **(F)** TDP-43 protein levels in the cytoplasm were quantified, and the mean values of TDP-43 protein levels in the sham group were normalized to 1.0. β-tubulin served as a loading control. All values are mean ± SEM, *p < 0.05 vs. sham; **p < 0.01 vs. ICH + empty vector; ^###^*p* < 0.001, ^##^*p* < 0.01, ^#^*p* < 0.05 vs. ICH + empty vector; ^@@^*p* < 0.01, ^@^*p* < 0.05 ICH + TDP-43 plasmid vs. ICH + TDP-43 plasmid mutation; ^&^*p* < 0.05 ICH + siRNA vs. ICH + control RNA. *n* = 6. **(G)** Immunofluorescence staining to assess distribution of TDP-43 after exposure to the TDP-43 plasmid mutation, and the brain region used for the slides was between the cerebral cortex and the perihematomal, scale bar: 50 μm.

To study effects of TDP-43 after ICH, we performed knockdown, overexpression, and mutation of TDP-43 using transfected plasmids or siRNA. The results showed that P-TDP-43 protein levels in ICH rats were significantly higher than in the sham group (Figures 3A–D). Compared with the wild type (WT) group, P-TDP-43 levels in the TDP-43 mutation group were significantly decreased. In addition, the siRNA group also showed a decrease in levels of *d* TDP-43 and P-TDP-43 (Figures 3A–D). Furthermore, to characterize further the subcellular distribution of TDP-43, we separated nucleoproteins and cytoplasmic proteins of TDP-43 using a nucleoprotein extraction kit. With the TDP-43 mutation at S409/410, protein levels of TDP-43 in the nucleus were significantly higher than those in the WT group (Figures 3A,E), while an opposite change was observed for TDP-43 levels in the cytoplasm (Figures 3A,F). Immunofluorescence staining confirmed these results, which showed that TDP-43 levels were elevated and decreased in the WT and siRNA groups, respectively, compared with their corresponding control group (Figure 3G).

### TDP-43 Mutations at S409/410 Attenuated SBI Following ICH

**Figure 4:**
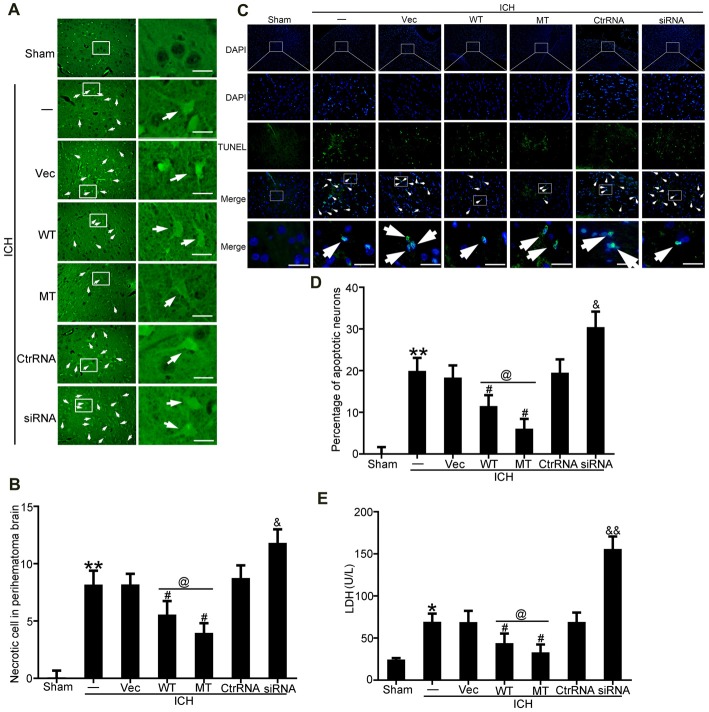
TDP-43 mutationattenuated SBI after ICH. **(A)** Fluoro-Jade B (FJB)staining was used to detect neuronal necrosis in the cerebral cortex.**(B)** Percentage of FJB-positive cells was calculated,and data are presented as mean ± SEM (**p < 0.01 vs. sham; ^#^*p* < 0.05 vs. ICH + vehicle; ^&^*p* < 0.05 ICH + siRNA vs. ICH + control RNA; ^@^*p* < 0.05 ICH + WT vs. ICH + MT), arrows indicate the positive neurons, scale bar: 50 μm. **(C)** TUNEL (green) counterstained with DAPI (blue) was performed, and thebrain region used for the slides was between the cerebral cortex andthe perihematomal. Arrows indicate apoptotic TUNEL-positive cells.**(D)** Percentage of TUNEL-positive cells. Data are presented as mean ± SEM (**p < 0.01 vs. sham; ^#^*p* < 0.05 vs. ICH + vehicle; ^&^*p* < 0.05 ICH + siRNA vs. ICH + control RNA; ^@^*p* < 0.05 ICH + WT vs. ICH + MT), arrows indicate the positive neurons, scale bar: 50 μm. **(E)** Lactate dehydrogenase (LDH) release in serum was detected by an LDH kit. Data are presented as mean ± SEM (*p < 0.05 vs. sham, **p < 0.01 vs. sham; ^#^*p* < 0.05 vs. ICH + vehicle; ^&^*p* < 0.05 ICH + siRNA vs. ICH + control RNA, ^&&^ *p* < 0.01 ICH + siRNA vs. ICH + control RNA; ^@^*p* < 0.05 ICH + WT vs. ICH + MT).

FJB and TUNEL staining were used to evaluate neuronal necrosis and apoptosis, respectively. The numbers of FJB-positive cells in the ICH group were significantly increased compared with the sham group. However, TDP-43 overexpression and mutation of its phosphorylation sites could inhibit the increase in number of necrotic cells induced by ICH. In addition, the protective effects were more pronounced in the mutation (MT) group than in the WT group (Figures 4A,B). However, the numbers of necrotic cells were increased significantly in the ICH + TDP-43 siRNA group compared with the Ctrl RNA group (Figures 4A,B). Next, we assessed neuronal apoptosis in rat brain samples using TUNEL (Figures 4C,D). Samples from the ICH group demonstrated histological evidence of apoptosis compared with the sham group, whereas TDP-43 overexpression and mutation groups showed decreased numbers of TUNEL-positive cells after ICH. Moreover, the MT group showed significantly decreased numbers of TUNEL-positive cells compared with the WT group (Figures 4C,D). In contrast, the group pretreated with ICH + TDP-43 siRNA showed an increase in the number of apoptotic cells in the rat brain sample (Figures 4C,D). We also measured LDH levels using an LDH detection kit and obtained results similar to those from FJB and TUNEL staining. TDP-43 mutations could inhibit LDH release, in contrast to an increase in release with TDP-43 knockdown (Figure 4E).

### TDP-43 Nuclear Loss Induced an Autophagy-Lysosomal Pathway That Could Be Blocked by Inhibiting mTORC1 Activity and Dynactin1 Expression After ICH

**Figure 5:**
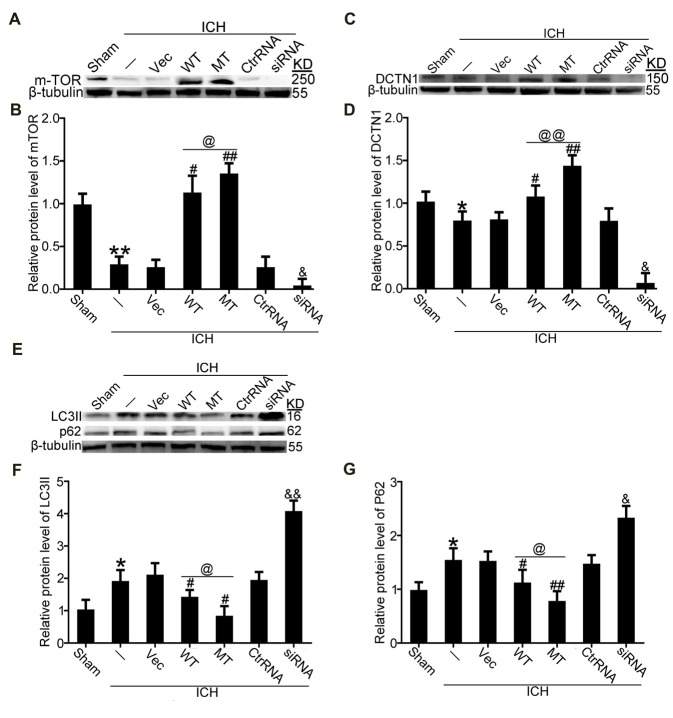
TDP-43 phosphorylation promoted biogenesis of autophagosomes and lysosomes and impaired autophagosome-lysosome fusion by inhibiting mTORC1 activity and dynactin1 expression after ICH. **(A)** Western blot analysis of mTOR protein levels after the indicated intervention. **(B)** mTOR protein levels were quantified, and the mean values of mTOR protein levels in the sham group were normalized to 1.0. β-tubulin served as a loading control. Data are presented as mean ± SEM (**p < 0.01 vs. sham; ^##^*p* < 0.01, ^#^*p* < 0.05 vs. ICH + empty vector; ^@^*p* < 0.05 ICH +TDP-43 plasmid vs. ICH + TDP-43 plasmid mutation; ^&^*p* < 0.05 ICH + siRNA vs. ICH + control RNA). **(C)** Western blot analysis of dynactin1 protein levels after the indicated intervention. **(D)** Dynactin1 protein levels were quantified, and the mean values of dynactin1 protein levels in the sham group were normalized to 1.0. β-tubulin served as a loading control. Data are presented as mean ± SEM (*p < 0.05 vs. sham; ^#^*p* < 0.05 vs. ICH + empty vector; ^@@^*p* < 0.01 ICH + TDP-43 plasmid vs. ICH + TDP-43 plasmid mutation; ^&^*p* < 0.05 ICH + siRNA vs. ICH + control RNA). **(E)** Western blot analysis of LC3II and p62 protein levels after the indicated intervention. **(F,G)** LC3II and p62 protein levels were quantified, and the mean values of protein levels in the sham group were normalized to 1.0. β-tubulin served as a loading control. Data are presented as mean ± SEM (*p < 0.05 vs. sham; ^##^*p* < 0.01, ^#^*p* < 0.05 vs. ICH + empty vector; ^@^*p* < 0.05 ICH +TDP-43 plasmid vs. ICH + TDP-43 plasmid mutation; ^&&^*p* < 0.01 ^&^*p* < 0.05 ICH + siRNA vs. ICH + control RNA).

It has been reported that TDP-43 can participate in autophagosomal and lysosomal biogenesis as well as in autophagosome-lysosome fusion in some neurodegenerative diseases (She et al., 2014; Schwenk et al., 2016). In this study, brain tissues were used to elucidate mechanisms of TDP-43 nuclear loss-induced SBI after ICH. In *in vivo* experiments, we found that protein levels of mTOR, which reflect activity of mTORC1, were significantly decreased in the ICH groups compared with the sham group. Nevertheless, compared with the ICH group, mTOR levels were significantly elevated after TDP-43 overexpression, and the TDP-43 mutation group showed higher levels than the WT group. However, the TDP-43 knockdown group showed an obvious decreasing trend when compared with the Ctrl RNA group after ICH (Figures 5A,B). Next, protein levels of dynactin1, which affect autophagosome-lysosome fusion, were assessed by western blot analysis. We found that the TDP-43 mutations could also prevent the decrease in dynactin1 expression induced by ICH when compared with the WT group (Figures 5C,D). Moreover, levels of autophagy related proteins, LC3 and p62, were measured by western blot. There was a remarkable increase in levels of LC3II and p62 in the ICH group compared with the sham group. With the TDP-43 mutation, which could inhibit phosphorylation of TDP-43, levels of autophagy were reduced compared with the TDP-43 WT group (Figures 5E–G).

### TDP-43 Mutations at S409/410 Ameliorated Neuronal Injury Induced by OxyHb

**Figure 6:**
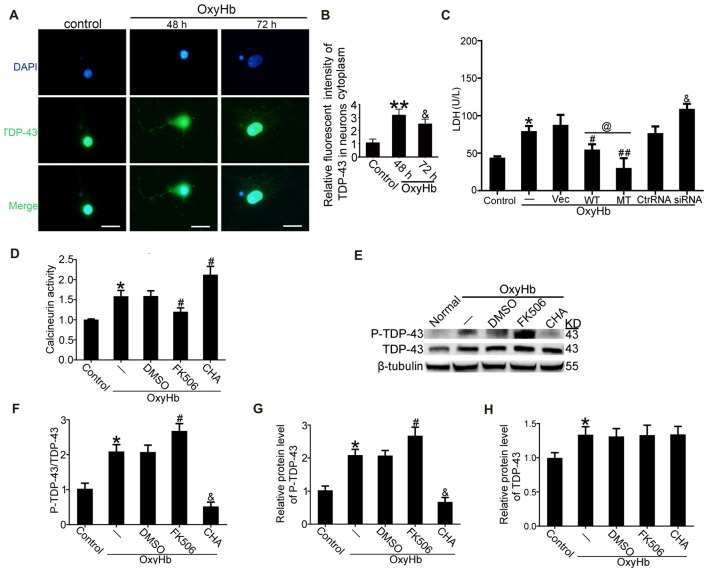
TDP-43 mutation reduced neuronal injury, and CN dephosphorylated TDP-43 after OxyHb treatment. **(A,B)** Immunofluorescence staining to assess changes in TDP-43 expression at 48 h *in vitro*. All values are mean ± SEM, **p < 0.01 48 h vs. sham, ^&^*p* < 0.05 48 h vs. 72 h, scale bar: 50 μm. All the experiments above were performed three times. **(C)** LDH release in serum was measured using an LDH kit. Data are presented as mean ± SEM (**p < 0.05 vs. control; ^#^*p* < 0.05, ^##^*p* < 0.05 vs. OxyHb + vehicle; ^@^*p* < 0.05 OxyHb + wild type (WT) vs. OxyHb + MT; ^&^*p* < 0.05 OxyHb + siRNA vs. OxyHb + control RNA). **(D)** Activity of CN after CHA and FK506 treatment was measured. Data are presented as mean ± SEM (*p < 0.05 vs. control; ^#^*p* < 0.05 OxyHb + DMSO vs. OxyHb + FK506 and OxyHb + CHA). **(E)** Western blot analysis of TDP-43 and P-TDP-43 protein levels after CHA and FK506 treatments. **(F)** P-TDP-43 protein levels were normalized to that of TDP-43. **(G)** P-TDP-43 protein levels were normalized to that of β-tubulin. **(H)** TDP-43 protein levels were normalized to that of β-tubulin. Data are presented as mean ± SEM (*p < 0.05 vs. control; ^#^*p* < 0.05 OxyHb + DMSO vs. OxyHb + FK506; ^&^*p* < 0.05 OxyHb + CHA vs. OxyHb + FK506).

To mimic ICH *in vitro*, neurons were treated with 10 μM OxyHb. Immunofluorescence staining was used to assess TDP-43 expression in neurons after OxyHb treatment. After OxyHb treatment, TDP-43 translocated from the nucleus to the cytoplasm. Compared with the control group, the shift peaked at 48 h and then rebounded by 72 h after OxyHb treatment, which was similar to results of the *in vivo* experiments (Figures 6A,B). Furthermore, we performed knockdown, overexpression, and mutation of TDP-43 by transfecting neurons with plasmids or siRNA, which was followed by OxyHb treatment. Afterward, cell culture supernatant was collected for measurement of LDH activity. Similar to the results from the *in vivo* experiments, TDP-43 mutations could inhibit LDH release, in contrast to an increase in release with TDP-43 knockdown (Figure 6C).

### P-TDP-43 Was Dephosphorylated by CN

Previous studies have indicated that CN can dephosphorylate P-TDP-43 (Liachko et al., 2016). Therefore, we examined the role of CN in phosphorylation of TDP-43 following ICH. CN agonist CHA and CN antagonist FK506 were used, and their expected effects were verified. Activity of CN clearly increased in the group treated with OxyHb when compared with the control group. Under OxyHb treatment, CN activity was significantly increased by CHA and decreased by FK506 (Figure 6D). Moreover, CHA was shown to weaken OxyHb-induced phosphorylation of TDP-43, whereas FK506 had the opposite effect (Figures 6E–H).

## Discussion

Numerous recent studies have shown that phosphorylated TDP-43 plays an important role in formation of UPIs in FTLD and ALS (Fatima et al., 2015; Nonaka et al., 2016). However, there have been few reports on the effects of TDP-43 in ICH-induced SBI. In this study, we demonstrated that ICH induced redistribution of TDP-43 from the nucleus to the cytoplasm both *in vivo* and *in vitro*, resulting in neuronal apoptosis and SBI. It is known that phosphorylation of TDP-43 affects its subcellular localization in part by formation of aggregates that prevent its return to the nucleus in both ALS and FTLD (Nonaka et al., 2016). Similarly, in our ICH model, we found that phosphorylation levels of TDP-43 increased significantly after ICH and peaked at 48 h, resulting in translocation of TDP-43 from the nucleus to the cytoplasm.

There are multiple potential phosphorylation sites within TDP-43, and S409/410 plays the most critical role (Neumann et al., 2009). Mutation of TDP-43 was performed both *in vivo* and *in vitro*, and we found that levels of P-TDP-43 were significantly reduced after mutations at S409/410. Furthermore, these mutations reduced nuclear loss of TDP-43. In addition, with the mutations of TDP-43, ICH-induced SBI was ameliorated as shown by decreased neuronal necrosis and apoptosis as well as reduced LDH release. However, TDP-43 knockout increased ICH-induced SBI, which is similar to effects of TDP-43 in ALS, showing that neuronal loss of TDP-43 can induce FTLD (Yousef et al., 2017). Also, TDP-43 knockout is lethal in mice, which indicates that TDP-43 in the nucleus is necessary for cell survival (Gendron and Petrucelli, 2011). Thus, it is reasonable that TDP-43 knockdown worsens ICH-induced SBI.

This study revealed roles of mTORC1 dysfunction as well as abnormal autophagy and lysosomal biogenesis in TDP-43-mediated neurodegeneration (She et al., 2014; Schwenk et al., 2016). In TDP-43-depleted cells, the distribution of mTOR in cellular localization experiments showed a dramatic reduction that reflects decreased activity of mTOR. Biogenesis of autophagosomes and lysosomes shows an opposite trend compared with activity of mTOR (Xia et al., 2016). Here, it was shown that inhibition of mTOR activity induced by ICH could be attenuated by the mutation that inhibited TDP-43 phosphorylation, resulting in a decrease in biogenesis of autophagosomes and lysosomes. Interestingly, dynactin1 was reported to affect autophagosome-lysosome fusion (Hwang et al., 2016; Tey et al., 2016). In our study, we found that ICH-induced reduction in dynactin1 was elevated by TDP-43 overexpression and mutation. In addition, it was found that autophagosome-lysosome fusion was blocked after ICH, but it could be promoted by TDP-43 overexpression or mutation. Nevertheless, with the loss of TDP-43, mTOR activity and dynactin1 expression were decreased more, which resulted in blocking autophagy flux. As indicated, TDP-43 is involved in autophagic cell death, and loss of TDP-43 leads to incomplete autophagy flux (Budini et al., 2017).

Since CN can dephosphorylate P-TDP-43 (Liachko et al., 2016), we examined the role of CN in phosphorylation of TDP43 following ICH. CHA has been shown to be a specific activator of CN (Hwang et al., 2016), and FK506 was used as a CN inhibitor to study the role of CN signaling in microcystin-LR triggered neuronal toxicity (Yin et al., 2009). In our study, we found that CN activity was increased in neurons after OxyHb treatment, and CHA and FK56 increased and decreased, respectively, CN activity after OxyHb-induced ICH. After CHA and FK506 treatment, TDP-43 phosphorylation levels were reduced and elevated, respectively. However, changes in neuronal apoptosis after treatment with CHA or FK506 will be examined in future studies. In addition, further pharmacological and toxicological experiments for CHA and FK506 are needed to assess potential side effects.

**Figure 7:**
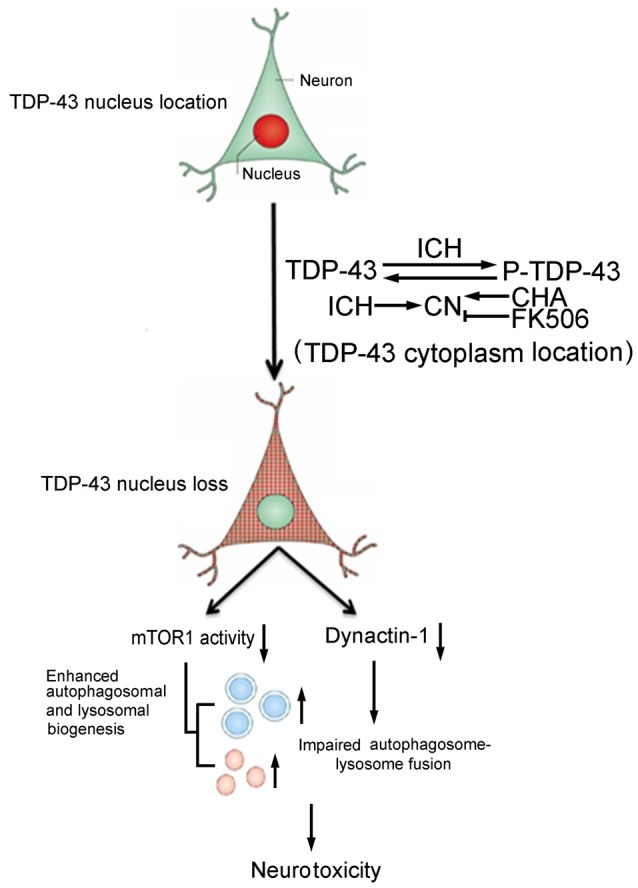
Schematic diagram illustrating possible mechanisms underlying phosphorylation and translocation of TDP-43 in SBI after ICH. After ICH, TDP-43 translocates from the nucleus to the cytoplasm and is phosphorylated, resulting in mislocalization of TDP-43. After nuclear loss of TDP-43, protein levels of mTOR and dynactin1 decrease, which leads to an increase in the biogenesis of autophagosomes-lysosomes and impairment of autophagosome-lysosome fusion. As a result, neurotoxicity and second brain injury occur. With an increase in P-TDP-43, activity of CN that can dephosphorylate TDP-43 increases after ICH. CHA can increase activity of CN and reduce levels of P-TDP-43, whereas FK506 has the opposite effect.

In conclusion, this study provided new insight into the cellular mechanisms underlying TDP-43 nuclear loss-induced brain injury after ICH. Phosphorylation-induced TDP-43 translocation may enhance biogenesis of autophagosomes and lysosomes and partly block fusion of autophagosomes and lysosomes, resulting in ICH-induced SBI (Figure 7). Thus, TDP-43 may be a potential therapeutic target for treatment of ICH-induced brain injury.

## Author Contributions

GC and ZY contributed to the conception of this study and designed the research. LS and KZ were responsible for conducting the experiments about the animal model and writing the manuscript. WZ and HS conducted the experiments about the cell culture. HL and HS helped to design the study’s analytic strategy and perform the analysis with constructive discussion. All authors have critically revised the manuscript and approved the final version.

## Conflict of Interest Statement

The authors declare that the research was conducted in the absence of any commercial or financial relationships that could be construed as a potential conflict of interest.
